# Teaching the Science in Neuroscience to Protect From Neuromyths: From Courses to Fieldwork

**DOI:** 10.3389/fnhum.2021.718399

**Published:** 2021-09-28

**Authors:** Alejandra Carboni, Alejandro Maiche, Juan C. Valle-Lisboa

**Affiliations:** ^1^Centro de Investigación Básica en Psicología e Instituto de Fundamentos y Métodos, Facultad de Psicología, Universidad de la República, Montevideo, Uruguay; ^2^Centro Interdisciplinario de Cognición Para la Enseñanza y el Aprendizaje, Universidad de la República, Montevideo, Uruguay; ^3^Sección Biofísica y Biología de Sistemas, Facultad de Ciencias, Universidad de la República, Montevideo, Uruguay

**Keywords:** neuroscience of education, learning, cognitive neuroscience, fieldwork, neuromyths, teacher training

## Abstract

In recent decades, Cognitive Neuroscience has evolved from a rather arcane field trying to understand how the brain supports mental activities, to one that contributes to public policies. In this article, we focus on the contributions from Cognitive Neuroscience to Education. This line of research has produced a great deal of information that can potentially help in the transformation of Education, promoting interventions that help in several domains including literacy and math learning, social skills and science. The growth of the Neurosciences has also created a public demand for knowledge and a market for neuro-products to fulfill these demands, through books, booklets, courses, apps and websites. These products are not always based on scientific findings and coupled to the complexities of the scientific theories and evidence, have led to the propagation of misconceptions and the perpetuation of neuromyths. This is particularly harmful for educators because these misconceptions might make them abandon useful practices in favor of others not sustained by evidence. In order to bridge the gap between Education and Neuroscience, we have been conducting, since 2013, a set of activities that put educators and scientists to work together in research projects. The participation goes from discussing the research results of our projects to being part and deciding aspects of the field interventions. Another strategy consists of a course centered around the applications of Neuroscience to Education and their empirical and theoretical bases. These two strategies have to be compared to popularization efforts that just present Neuroscientific results. We show that the more the educators are involved in the discussion of the methodological bases of Neuroscientific knowledge, be it in the course or as part of a stay, the better they manage the underlying concepts. We argue that this is due to the understanding of scientific principles, which leads to a more profound comprehension of what the evidence can and cannot support, thus shielding teachers from the false allure of some commercial neuro-products. We discuss the three approaches and present our efforts to determine whether they lead to a strong understanding of the conceptual and empirical base of Neuroscience.

## Introduction

The rapid development of neurosciences in the last few decades motivated the search for applications of this body of knowledge and an increase of the interest of the public (Herculano-Houzel, 2002; Altimus et al., 2020). As any advanced field, Neuroscience is complex, thus the potential for excessive simplifications, tergiversation or even outright falsification is high. The appearance of Neuroscience in the public discourse led to the emergence of several neuromyths that spread through the population. The natural interest of the educators in Neuroscience sparked the development of a market of several products aimed at educators and parents that were supposedly based on Neuroscience, but the support is scarce. Early on, in 1997, John Bruer (1997) published his now classical paper “Education and the brain: A bridge too far” pointing to several holes in the Neuro-educational literature and products, and promoting a skeptical (but hopeful) view on the then available application of Neuroscience to Education. Also, Bruer suggested that there was indeed an available bridge in Cognitive and Educational Psychology having the required body of knowledge to impact on Education. Despite making an instant classic, the paper did not stop the neuromarketing of dubious ideas that helped promote several wrong or simplified ideas, usually called neuromyths.

The concept of neuromyth refers to a series of misconceptions or baseless beliefs that arise from the wrong interpretation of neuroscience research results and its application in education or other contexts (OECD, 2002). Several factors related to the emergence and proliferation of neuromyths have been identified: differences in training and technical language between the educational and neuroscientific fields (Howard-Jones, 2014), limited access to peer-reviewed scientific journals (Ansari and Coch, 2006), overgeneralization from neuroscience studies with individual neurons to educational policy (Goswami, 2006), and preference for explanations that seem based on scientific evidence even though there is no evidence in this regard (McCabe and Castel, 2008; Weisberg et al., 2008).

One aspect of the lists of neuromyths used in several papers (Herculano-Houzel, 2002; Dekker et al., 2012; Gleichgerrcht et al., 2015) is that they are variable in their character. For instance, the difficulty of the questions in the Neuromyth scale (Howard-Jones et al., 2009; Dekker et al., 2012) is variable, as should be if the scale is to measure anything. More important to us here, the questions are of very different types. Consider for instance, these 4 questions: (1) *We mostly only use 10% of our brains*, (2) *Drinking* < *6–8 glasses of water a day can cause the brain to shrink*, (3) *Keeping a phone number in memory until dialling, recalling recent events and distant experiences, all use the same memory system*, (4) *Memory is stored in the brain much like as in a computer. That is, each memory goes into a tiny piece of the brain*. Neuromyth (1) is so imprecise that it could be argued that it is not even false, it is unscientific. Neuromyth (2) is easily spotted as false with usual experience; Neuromyth (3) contradicts a detailed and important piece of knowledge that required the careful study of patients and experimental studies during several years to be established (Squire, 2009). Lastly, neuromyth (4) can be said to be defended by some well known cognitive scientists (Gallistel and King, 2009) and even has some empirical support (Johansson et al., 2014).

Thus in a sense the list of neuromyths can change at any time, and maybe some of the less improbable assertions can be re-interpreted under a new framework. The problem is not so much having wrong beliefs about the brain; after all in a growing science there has to be some level of controversies that are part of “normal science” (Kuhn, 1962). The problem is that while professional scientists can gauge the evidence base of any claim and search for the relevant evidence to rule out some assertion, non-professionals are at the mercy of the best communicators, not necessarily the most truthful.

We believe that the only way out of this problem is to develop strategies to teach scientific thinking, that is to teach the public, and specifically the Educators, how scientists deal with the different opinions around a set of propositions about a body of knowledge. Here we describe our attempts to develop a strategy and a preliminary evaluation of the success of the different alternatives. First we describe the origin of our proposal. After describing the basis for our strategies we present the evaluations that show that teacher directed courses about the theoretical bases of Cognitive Neuroscience and the participation within research groups are viable strategies to give a rigorous science education. We create a questionnaire to assess the teacher's knowledge of epistemological principles, and show that the pattern of responses suggests that although teachers that take part in research groups are not better at answering the neuromyth questionnaire than those that took our teacher directed course, some of them show signs of thinking similarly to researchers. Despite the limitations of the evaluation, we believe that participation in research can help teachers develop a scientific mindset which allows them to better navigate the specialized literature and the commercial offerings.

In the last few years we have been leading a set of projects aimed at the development of the Science of Learning and its applications to Education. We were part of the organizing and steering committee of the Latin American School for Education, Cognitive and Neural Sciences, a summer school that had 7 editions: three times in Chile, two times in Argentina, one in Brazil and one in Uruguay (for the Uruguayan edition see, http://2014.laschool4education.org). These Summer Schools brought together consolidated researchers on the Science of Learning (Meltzoff et al., 2009; Ansari, 2021) together with graduate students or junior PIs in order to further the development of application of this nascent field to Education. It was born as the result of a meeting that took place in Santiago de Chile in 2007, which brought together scientists interested in the Brain/Education barrier and led to the Santiago Declaration (https://www.jsmf.org/santiagodeclaration/), in part a re-evaluation of Bruer's paper main thrust.

Judging not only by the opinion of the alumni and Faculty involved, but also from the standpoint of the collaborative projects and publications that the Schools promoted, these instances have been a great success launching a series of studies in our region (Goldin et al., 2014; Sigman et al., 2014; Strauss et al., 2014; Dillon et al., 2015; Odic et al., 2016; Valle-Lisboa et al., 2016). Likewise, it has created a scientific community with high dedication to the popularization of this new Science^1^ especially to educators^2^.

Motivated by the environment and international collaborations that were forged in LASchools, each group of researchers have been taking different perspectives for the implementation in their countries of that interface between education and cognitive science. Thus, in Uruguay, together with a group of colleagues from Neuroscience, Psychology and Computer Science, we created in 2015 the Interdisciplinary Center for Cognition for Teaching and Learning (https://www.cicea.ei.udelar.edu.uy/) and in 2016 the first master in cognitive science in Uruguay (https://www.mcc.ei.udelar.edu.uy/) with a marked profile towards topics related to the Learning Sciences.

In this framework, in 2017 we launched the first symposium of education, cognition and neuroscience that brings together some consolidated researchers from the LASchool's environment together with researchers, educators and policy decision makers. Besides the researchers and students that attend the symposium, more than 400 educators attend to the symposium motivated by their interest in the possibilities that this new^3^ interface between education and cognitive science could offer. The result was a 4-day event where international speakers (such as Manuel Carreiras, Justin Halberda, Sidarta Ribeiro, Mariano Sigman, Linda Smith) presented their latest results and participated in round tables together with local policy makers analyzing questions such as “What contributions can Cognitive Science make to Education in Uruguay ” or “What is the kind of University of Education that Uruguay needs? “ All these instances of the Symposium are available on the YouTube channel^4^ of CICEA.

In parallel to these singular activities^5^, in Uruguay we have been organizing annually (since 2018) a course directed to teachers and educators (https://www.cicea.ei.udelar.edu.uy/curso-aportes-de-las-ciencias-cognitivas-a-la-educacion-2/) with the main idea to show the fundamental principles of cognitive neuroscience, with the specific objective of discussing the impacts the new Science of Learning has on the theories they use to guide their practice. With this idea in mind, the course goes over general principles of cognition, learning, teaching, plasticity, motivation and also some specific topics of this interface like math cognition and language. This course brings annually more than 200 educators so it could be considered part of the permanent link that educators in Uruguay have with the research and advances of cognitive sciences and learning sciences.

Lastly we started a new, but more costly effort, namely, organizing scientific stays for teachers to do research applied to education in our labs. In this way, a limited number of educators have become progressively approaching the different research groups that work in Uruguay on these topics. These educators have been integrated in research groups by providing their experience and their links with the Educational System at the same time that they participate in some of the research projects that these groups develop. This experience has been novel and challenging since it has allowed us to see the difficulties of interdisciplinary work in practice. However, most researchers evaluate it as a positive experience although it is still premature to draw conclusions about results.

We conceive our efforts in three levels or strategies. In the first place, our popularization efforts, or scientific symposia where teachers are invited to participate. The second strategy is the yearly course on Cognitive Science and Education. The third strategy is the organization of research stays for teachers. The first strategy is defined by exposure to Neuroscience that might be relevant for Education, but only through popularization instances (magazine or newspaper articles, popularization talks, booklets, etc.) or by short symposia, that despite gathering important researchers, are too short to allow the transmission of a great deal of knowledge. The second strategy is clearly defined by the participation in any of the editions of our course of “Contributions of cognitive sciences to education.” This course includes lectures and paper discussion sessions, where participants are directed to analyze the methodology of the results presented. The third strategy implies taking part in research activities within any of the research groups of CICEA, for at least 1 month.

In this article we present our preliminary analysis of the impact these three strategies have in the teacher's knowledge of Cognitive Science and its applicability to Education. In order to approach this evaluation we ran two surveys. One survey is an adapted extract from the usual Neuromyth measuring scales (Howard-Jones, 2014). The other is a set of six questions related to general epistemological and methodological questions. As we will show, despite the fact that much more work needs to be done, these epistemological/methodological questions complement the neuromyth scale, adding a dimension related to procedural knowledge. We conclude by proposing that the core of understanding of Neuroscience in a non-specialist public, depends on a broader Scientific Education.

## Methods

### Participants

Previously to applying the survey, we defined four categories with which we categorized participants in four different groups. Group 1 (we named them, the Interested, group): included teachers from any education level interested in Cognitive Neuroscience but that at most might have attended popularization talks related to the topic (*N* = 48); Group 2 (Attended): is composed of teachers that participated in any of the editions of the course “Contributions of cognitive sciences to education” (Aportes de la ciencias cognitivas a la educación) (*N* = 60); Group 3 (Collaborated): included teachers that are taking part in any of our Educational projects as members of the research team (*N* = 11) and Group 4 (Researchers): composed of post graduate students and junior Investigators, which will serve as a gold standard (*N* = 18). The sample comprised 140 participants linked to education and cognitive neuroscience aged between 20 and 69 years old (mean age = 42.9, SD = 10.7), and was selected using the following criteria: first we contacted all the teachers (12) that were taking part in research activities. Only one did not answer the survey. In order to have a comparable “gold standard” we recruited all young researchers and graduate students from our lab. We also contacted all attendees to the two “Aportes” courses (192) who completed the evaluations during the course and recorded all the responses we received before we started to analyze the data (*N* = 60). We aimed for a similar number of teachers who did not attend any of our courses or seminars, nor took part in any other activity we organized. The survey was promoted through teachers' mail-lists, and through social networks. All interested participants were directed to an online survey (google form) and answered the questions anonymously. Participants completed a questionnaire allowing us to determine whether they fulfilled the inclusion criteria. Three subjects were excluded because their performance was more than 2.5 standard deviations from the mean score (see below, data analysis).

### Procedure

Participants were asked to answer through a Likert scale (1–5) the degree of agreement with 38 statements related to neuroscience and education. 32 assertions were selected and adapted from Howard-Jones et al. (2009) survey. Adaptation of the items involved straightforward improvements in the expression to support clarification in Spanish. Additionally, 6 more statements were included in order to evaluate general aspects related to epistemological investigation knowledge (please see Supplementary Material for all the questions used).

### Data Analysis

As we mentioned before, we sent surveys to four groups of people. We eliminated the data from subjects whose score differed from the global (i.e., considering all subjects irrespective of group) mean score by more than 2.5 global standard deviations; this resulted in the elimination of the data from three participants that had scores lower than the mean; according to the classification in groups, these participants were part of group 1.

#### Missing Data Imputation

We replaced missing values with the median of the responses for each item. Overall, we imputed <1.1% of the data.

In all analyses we used the scikit.learn python library for clustering analyses, pandas data frames and scipy.stats tools (statistical tests).

## Results

Following standard procedures, we eliminated the items whose correlations with the full score were negative. These negative correlations imply that some participants who get high scores, are getting low scores on those items, and some participants having overall low scores, answered those questions correctly. The removal of these items ensures that we only keep items that measure the same abilities as the rest.

The eliminated items were:

- 'El consumo regular de refrescos con cafeína reduce el estado de alerta', [Regular drinking of caffeinated soft drinks reduces alertness].
- 'Los alumnos muestran preferencias individuales sobre el modo en que reciben información (por ejemplo, visual, auditiva, cinestésica) '[Individual learners show preferences for the mode in which they receive information (e.g., visual, auditory, kinaesthetic) ].

As we inverted scores of the questions whose correct answer was 1 in the Likert scale, the maximum achievable score in the Neuromyth Scale after these manipulations was 150, the result of obtaining 5 points in each of the 30 remaining items. The Cronbach's Alpha obtained was χ = 0.68 which is acceptable for our purposes.

In Table 1 we show the scores for each group after all these manipulations.

**Table 1:** Group composition and descriptive parameters of the neuromyth survey results.

**Group**	**No. of members**	**Median score**	**Inter quartile interval**	**Q1**	**Q3**
Interested	48	109.5	10.5	105.5	116.0
Attended	60	115.5	11.0	111.0	122.0
Collaborated	11	116.0	8.5	111.5	120.0
Researchers	18	130.5	9.0	124.0	133.0

*Q1 and Q3 are the first and third quartile, respectively*.

**Figure 1:**
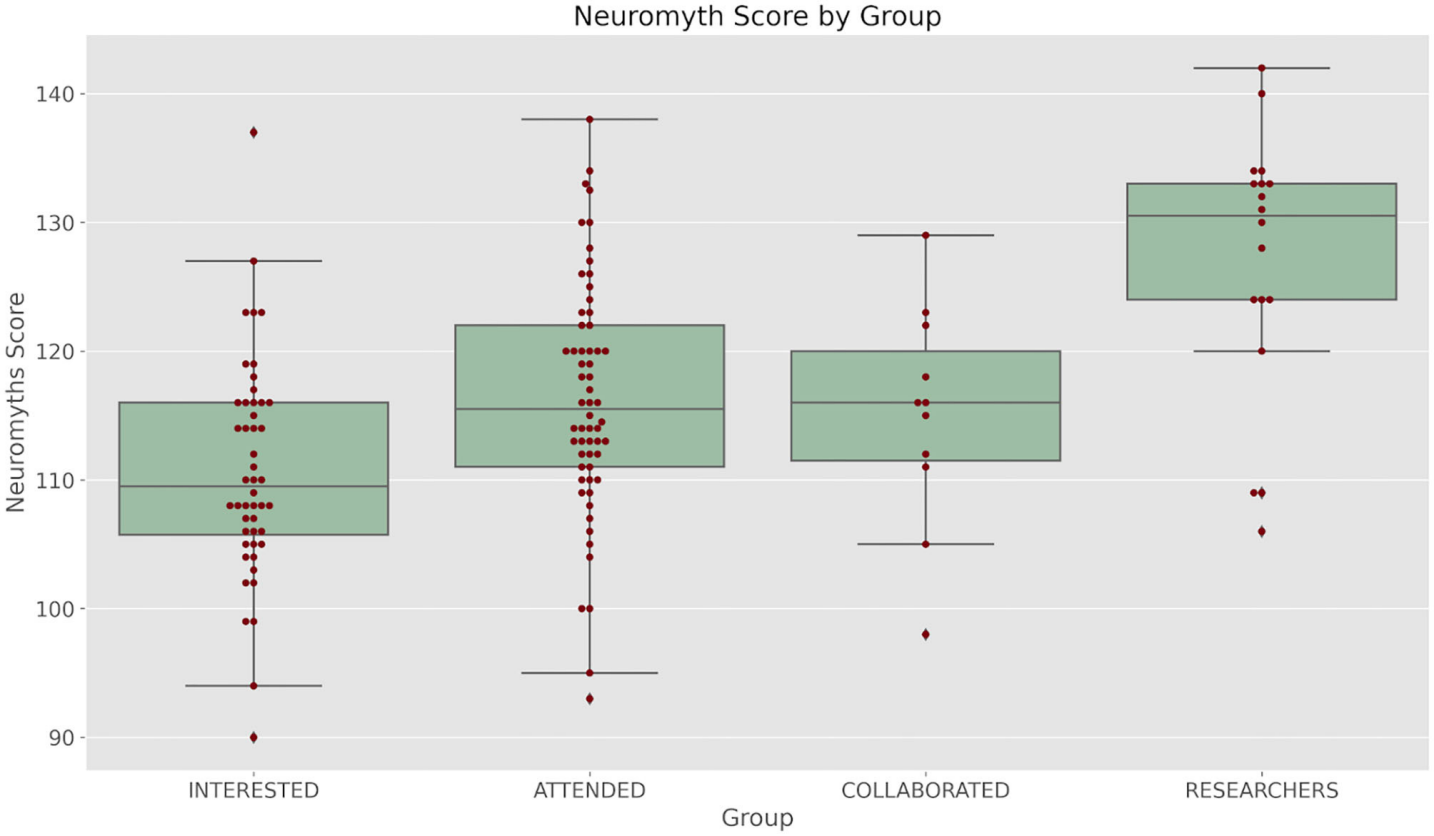
Boxplots showing the distributions of Neuromyth scale scores: INTERESTED refers to a group of teachers not related to our courses and stays. ATTENDED refers to a group of teachers that have participated in the regular course relating Cognitive Science and Education. COLLABORATED refers to those teachers that besides taking part in our course have done or are doing a research stay at our lab. RESEARCHERS Refers to Master students, PhD students or researchers from our lab. See the text for further details.

In Figure 1 we present the distribution of scores in the Neuromyths scale. By inspection it can be seen that the group of researchers is clearly separated from the other groups. It also seems that the second group has a higher median score than the first group. The third group is small and variable.

This intuition is confirmed by the analysis. A Kruskal-Wallis test shows that there is a statistically significant difference between the score distribution of the groups (*H* = 30.062, df = 3, *p* = 1.34e−06). The *post hoc* comparison using Dunn's test with false discovery rate correction shows that the scores of group 4 are different from all the other groups, that group 2 and group 3 are different from group 1 and that group 2 and 3 do not differ in their mean scores (Table 2).

**Table 2:** Dunn's test with False Discovery Rate (FDR) correction.

	**Interested**	**Attended**	**Collaborated**	**Researchers**
Interested	–	**0.00107**	0.0519	**1.80E−07**
Attended		–	0.267	**0.001073**
Collaborated			–	**0.006124**
Researchers				–

*The table shows the adjusted exact p-values (bold marks the significant differences at 0.01 level for ease of visualization) of each pairwise comparison between mean neuromyth scale scores of each group*.

### The Pattern of Responses

**Figure 2:**
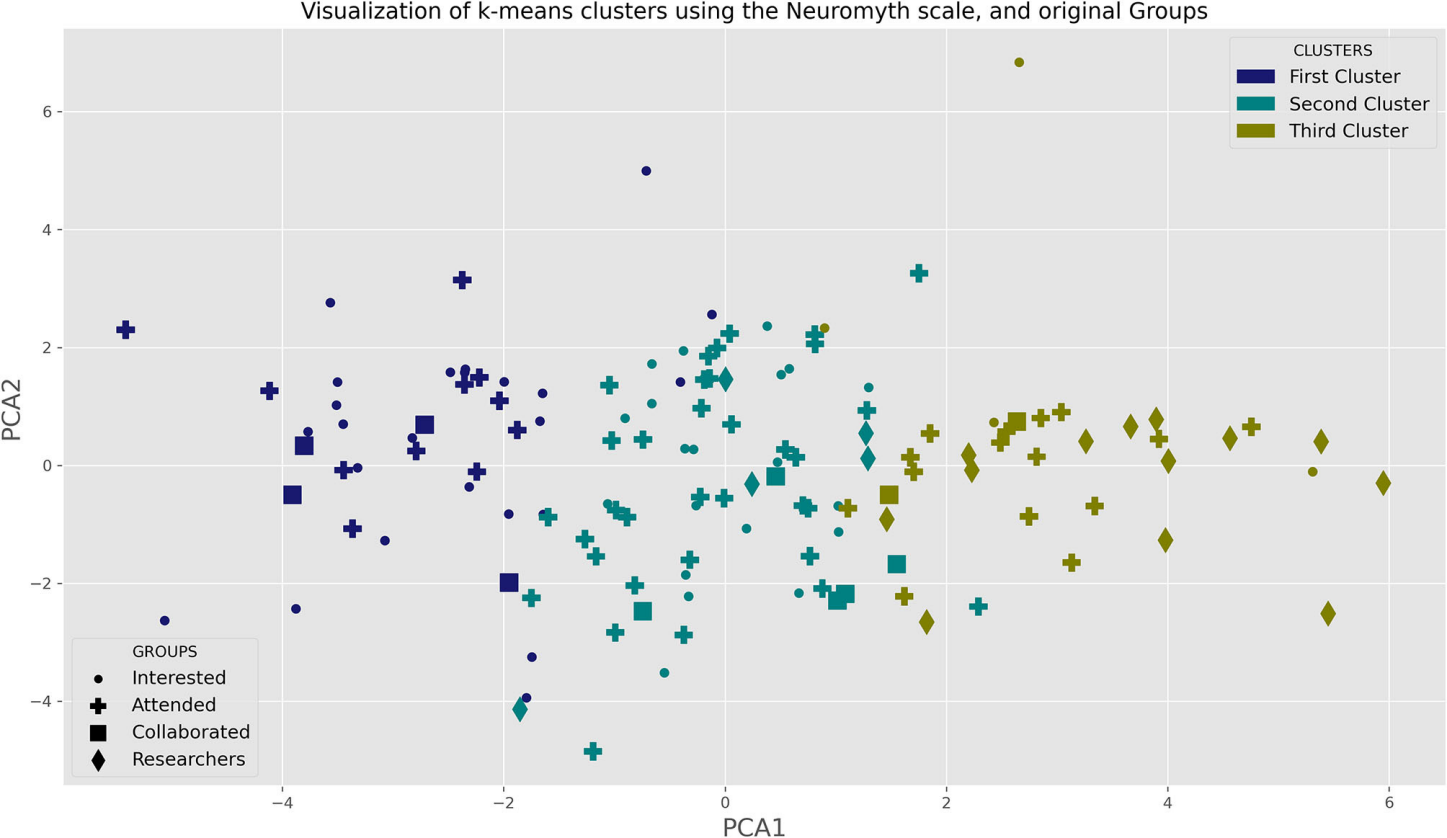
2D projection and clustering of the responses to the Neuromyth questionnaire. The responses to the Neuromyth Questionnaire were projected to the 2 dimensional space spanned by the principal components of responses. The full set of responses was used to cluster the respondents in three clusters using k-means algorithm. In the graph, the Groups are coded by the markers and the clusters obtained by k-means are coded using colors. Notice that some points seem to be misplaced, but this is due to the fact that the PCA does not capture enough variance (see Supplementary Material for details of the PCA results).

In order to deepen our understanding of the knowledge of each group, we turn to analyze the pattern of the responses. At the outset, we expected that the members of the fourth group would have a small variability in their responses. Surprisingly this is not the case. But this variability can be attributed to different patterns of response in each of the groups. In this sense, despite having the same overall scores, groups might differ in their pattern of responses. To analyze the patter of responses we first run a PCA with all the answers to the Neuromyth questionnaires. The first four principal components (PCs) only explain 39 % of the variance. To analyze the response pattern we clustered the four groups using k-means to fit three clusters to all the questionnaire responses. Despite the fact that PCA captures little variance, in order to visualized the clusters obtained with k-means, we project all the responses to the plane formed by the first two principal components. We show the results of the k-means clustering in the PCA plane in Figure 2, where we use a different marker for each group and a color for each cluster.

Even though PCA is not capturing enough variance, notice that aside from some exceptions the first PC separates the three groups quite well. This first PC correlates strongly with correct responses to the following questions (see Supplementary Material):

- *Short bouts of coordination exercises can improve integration of left and right hemispheric brain function*.
- *Environments that are rich in stimulus improve the brains of pre-school children*.
- *It is with the brain, and not the heart, that we experience happiness, anger, and fear*.

The second PC correlates with the correct response to:

- *Learning problems associated with developmental differences in brain function cannot be remediated by education*,

and with the incorrect response to:

- *Learning is not due to the addition of new cells to the brain*.
- *The mind is the result of the action of the spirit, or of the soul, on the brain*.

As a result, a high PC1 score and medium PC2 score is associated with most of the members of group 4 which are mostly in cluster three. Most of the individuals from group 1 are in the first cluster, whereas both group 2 and 3 are distributed between clusters 1, 2, and 3. In Table 3 we show the detailed clustering attribution.

**Table 3:** Results of clustering participants by their answers in the neuromyth scale.

	**Cluster 1**	**Cluster 2**	**Cluster 3**	**Total**
Interested	24 (50%)	20 (42%)	4 (8%)	48
Attended	11 (18 %)	34 (57 %)	15 (25 %)	60
Collaborated	4 (36 %)	5 (46 %)	2 (18 %)	11
Researchers	0	5 (28 %)	13 (72 %)	18
Total	39	64	34	137

*The clustering was obtained using the k-means algorithm, with 3 clusters. The distribution among clusters is different in the different groups (χ^2^ = 40.02, p = 4.52e−07). Post-hoc comparisons with FDR correction show that the cluster distribution of group Interested differs from that of group Attended (p = 0.0022), and from Researchers (p = 1.49× 10^−6^) but not from Collaborated (p>0.5). Attended also differs from Researchers (p < 0.003) but not from Collaborated (p > 0.4). Collaborated and Researchers also have different distributions among clusters (p = 0.0061)*.

Thus the same results as the comparison between total scores are apparent with the clustering method, i.e., group 4 and group 1 are extremely different, but both group 2 and 3 share part with group 1 and 4. In that sense, the pattern of responses is similar between groups 2 and 3.

### Methodological and Epistemological Questionnaire

**Figure 3:**
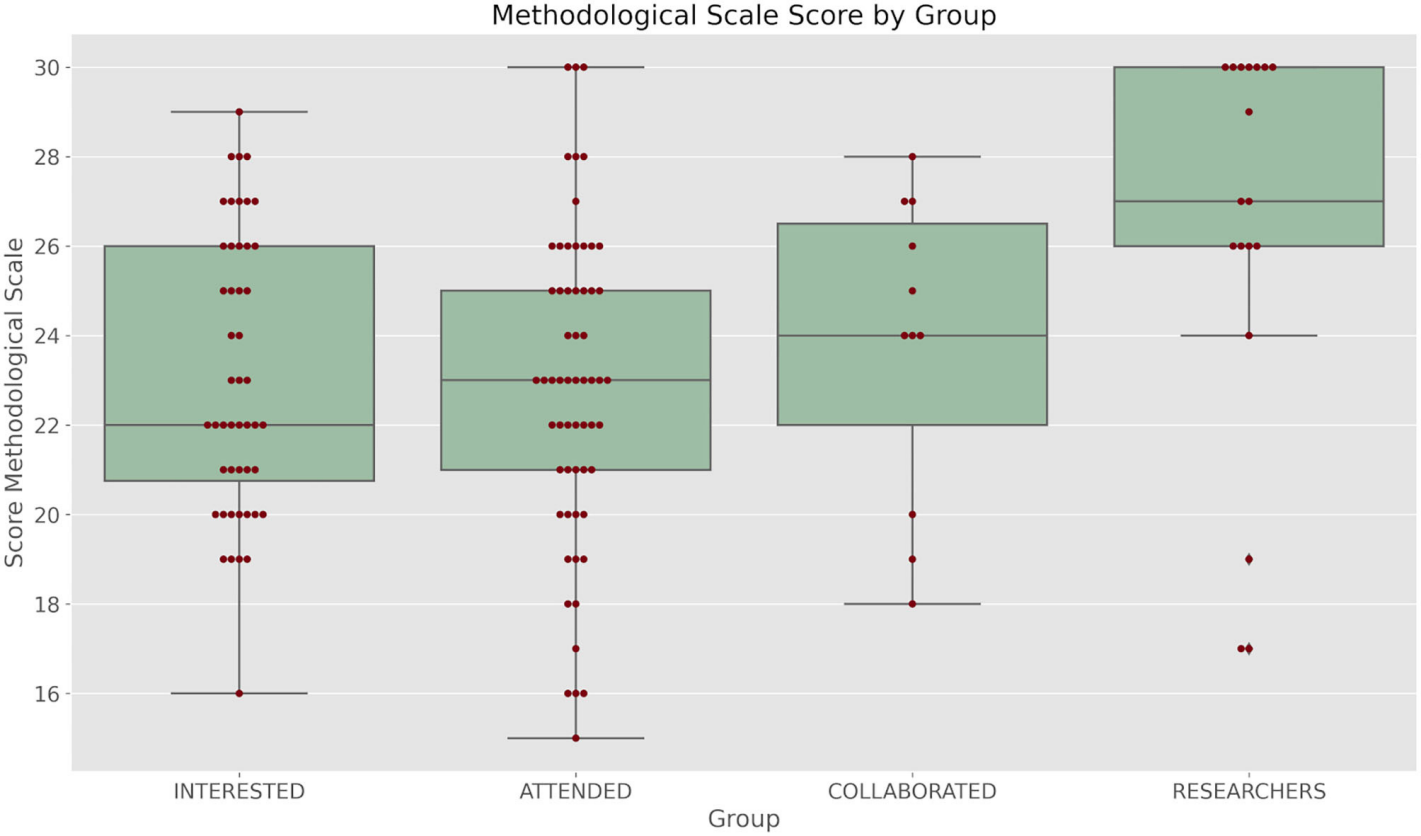
Boxplots of the scores obtained in the methodological/epistemological questionnaire by each group.

As a part of an ongoing strategy to analyze the epistemological and methodological knowledge of our students and collaborators, we applied a modified questionnaire adapted from our School of Psychology methodological undergraduate courses. The details of the questionnaire are shown in the Methodology section. In Figure 3 we show the scores obtained by each group in this questionnaire. A Kruskal-Wallis test shows that the scores differ (*H* = 12.82, *p* = 0.005).

A *post-hoc* Dunn test shows that only the scores from GROUP 4 and GROUP 1 (*p* = 0.0029) and GROUP 4 and GROUP 2 (*p* = 0.0026) differ. All other *p* are higher than 0.05.

**Figure 4:**
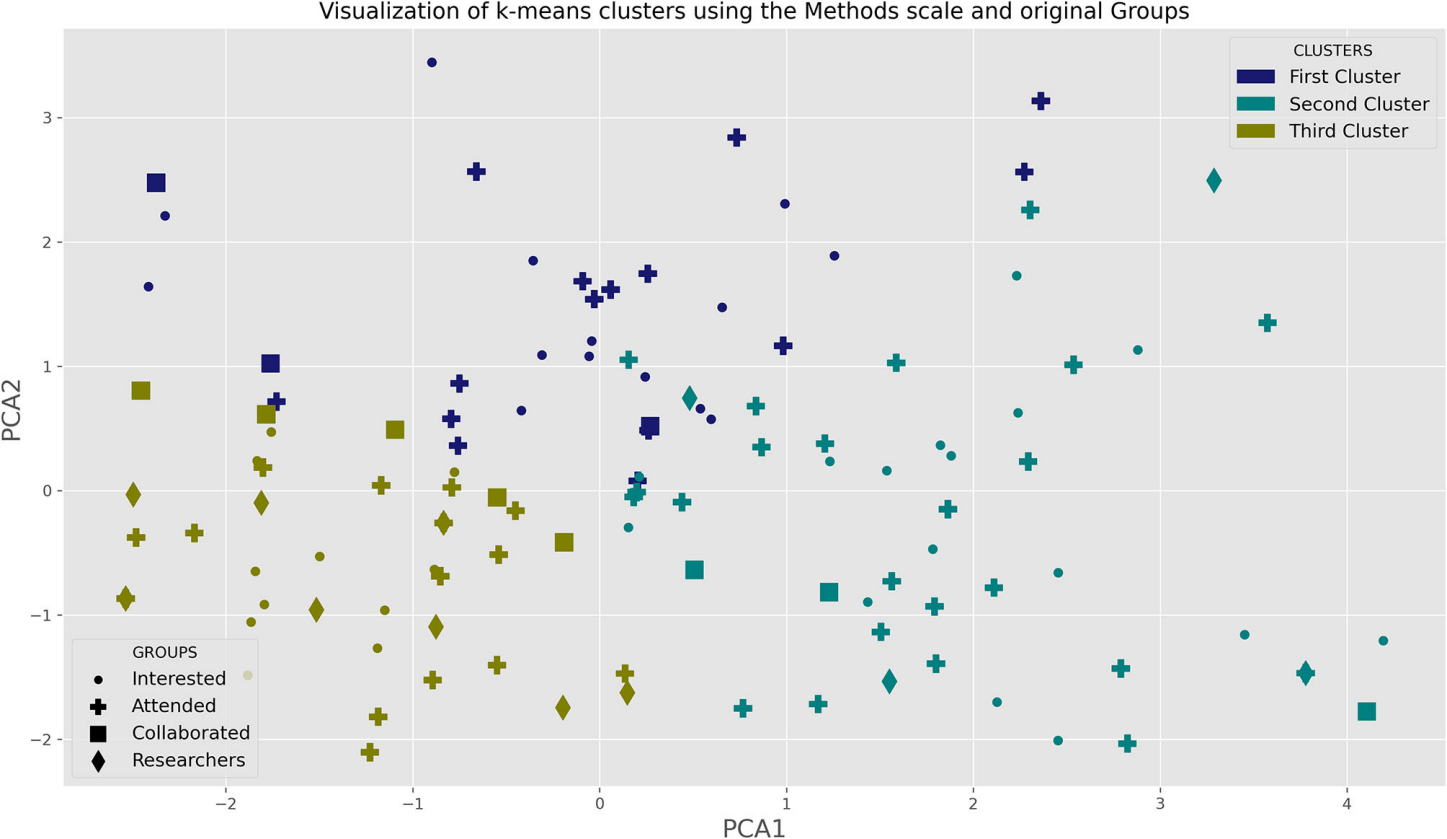
Results of k-means clustering algorithm, projected over the plane spanned by the two first PCA dimensions. The markers represent the groups, whereas the color represents the clusters. In the plane, the lower left corner is associated with the third cluster, the lower right with the second cluster and the upper center with the first cluster. Most members of the RESEARCHERS group (diamond) are in the third cluster, and just a few are in the second cluster with none in the first cluster. Both INTERESTED (points) and ATTENDED (crosses) are more evenly distributed, whereas COLLABORATED (squares), tends to be closer to the RESEARCHERS distribution (it is indistinguishable statistically, see Table 4); there are 5 squares in that cluster. Although due to low numbers it is also indistinguishable from the first two groups, notice that whereas in the clustering based on the Neuromyth scale, COLLABORATED and RESEARCHERS were statistically different (Table 3), here they are not.

We further use the pattern of responses to these six questions as clustering features following the procedures we applied to the other questionnaire. In Figure 4 we show the results of the clustering algorithm.

We used PCA to visualize the cluster obtained. Here the first two PCs capture 55 % of the variance.

The first PC correlates to the incorrect answer to these questions:

- *A primary school applies a method for teaching math, then takes an exam and all of its students pass. We cannot claim that the method is effective for teaching mathematics*
- *A Nobel prize winning researcher claims that the technique he developed many years ago makes it possible to assess whether a person is infected by a virus. This does not show that the technique can be used to assess whether a person is infected with a virus*.
- *A group of students summarize texts and perform well on tests. This proves that summarizing is a good way to study*.

The second PC correlates to the incorrect answers to these two questions:

- *In order to evaluate an initial literacy program, children are randomly selected from various classes in the country, dividing them into two statistically indistinguishable groups. One of the groups learn through the new program and the other through a regular program. If external evaluators find statistically significant advantages in those children who participated in the new program, it is possible to claim that it is more effective for initial literacy than the usual program*.
- *A researcher analyzes the relationship between a set of variables and reading scores. He finds that those children who have higher relative weight read better than those who have low weight. Therefore, the researcher shows that increasing weight improves reading*.

and to the correct answer to the questionnaire

- *A group of students summarize texts and perform well on tests. This proves that summarizing is a good way to study*.

See Supplementary Materials for details of the PCA.

In Table 4 we detail the participation of each group in each cluster. In the legend of this table, we analyze the statistical properties of the distribution.

**Table 4:** Clusters obtained from the pattern of responses to the methodological questionnaire.

**Group**	**Cluster 1**	**Cluster 2**	**Cluster 3**	**Total**
Interested	15 (31 %)	17 (36 %)	16 (33 %)	48
Attended	15 (25 %)	25 (42 %)	20 (33 %)	60
Collaborated	3 (27 %)	3 (27 %)	5 (45 %)	11
Researchers	0	4 (22 %)	14 (78 %)	18
Total	33	49	55	137

*The different groups are differentially distributed among clusters. (χ^2^ = 14.83, p < 0.05). Post hoc comparison with FDR correction shows that the group Interested and group Researchers are different (p < 0.01) as well as Attended and Researchers (p < 0.01). None of the other groups show statistically significant differences in their distributions among clusters (all ps > 0.05)*.

## Discussion

Neuroscience is one of the fastest growing sciences in the last decades, both in terms of papers published and of the impact it has on the public. This impact is not only due to the scientific advances in understanding the brain, but especially because it can give new insights into old and persistent problems. One of the areas where it is hoped that Neuroscience can greatly improve human life is in the field of Education. Indeed, several approaches towards basing Educational practices in Cognitive Neuroscience have been emerging in different places of the world. The initial reasonable skepticism (Bruer, 1997) has been replaced by optimistic approaches applying Cognitive Neuroscience to Education in reading (Hruby et al., 2011; Potier Watkins et al., 2019), mathematics (Halberda et al., 2008; Dillon et al., 2017; Judd and Klingberg, 2021), social skills learning (Gerdes et al., 2011), science education (Zimmerman and Klahr, 2018), motivation (Di Domenico and Ryan, 2017) attention (Stevens and Bavelier, 2012), conceptual development (Mareschal, 2016) and creativity (Onarheim and Friis-Olivarius, 2013) among others [but see Bowers (2016) for criticism of the neurobiological aspects of these approaches]. A few years ago, as we started participating in the regional effort to develop Cognitive Neuroscience and its applications to Education we sought to produce applications to Education. Together with the use of digital technology this allowed us to study and intervene in an educational setting (Valle-Lisboa et al., 2016).

Coupled to this renewed interest in Neuroscience, a commercial promotion of supposedly Neuroscience-based programs and products has been growing and promising several simplistic solutions to Educational problems and rebranding old strategies under a “neuro” slogan with the purpose of increasing the revenues. A group of false beliefs on the workings of the brain, called neuromyths, are widely spread and threaten to replace genuine deep knowledge in the population as a whole and, potentially more dangerously, within the teacher professionals.

From the beginning of our applied research projects we realized the importance of Education to counter these neuromyths and their impact on educators. Nevertheless, it has been shown that those teachers that are more informed about neuroscience tend to be more vulnerable to neuromyths (Dekker et al., 2012) probably because they are more exposed to low quality materials. It follows that any program geared at educating teachers about neuroscience has to be carefully designed in order to avoid unintendedly promoting neuromyths.

In this article we have shown that among all teachers and educators that are interested in Neuroscience, those that take part in our longer duration activities, be them courses or the participation in research stays, are less vulnerable to neuromyths than those that only attend our popularization talks or read popularization publications. Although the results presented come from a correlational study that was a spinoff of our efforts to establish a definite teaching strategy, we believe that this is not just due to differences in motivation. Indeed as a part of the initial questionnaire we asked participants about their interests and all participants declared their intention to apply Neuroscience to education. Moreover members of group 1 also attended other short talks or symposia, so we believe that there are no systematic differences in motivation between the three groups of teachers. Although we are starting an experimental study in order to clearly separate causal and non-causal effects, if the causal link is confirmed, it would suggest that the difference observed in our results might be caused by the participation in the long-lasting activities. This would not really be surprising. Most popularization activities only transmit a superficial explanation of the phenomena involved, and thus at the same time that they promote the interest in the topic, when they are not presenting the empirical basis of the claims, these activities do not promote a deep understanding of neuroscience. Our course involves over 30 h of lectures and paper discussions, connecting teachers to the fundamentals of the discipline. It is not surprising that participants in our course are better at responding to the questions, although the specific content of most of these questions is not taught directly in the classes. Interestingly, most of the teachers that decided to participate in our research teams also had a higher score in the Neuromyth scale than the general group of teachers. Although every instance of participation of teachers in research groups involved reading scientific articles, we did not make sure that all the educators read the same articles. Nevertheless, their responses did not differ from those of the group of teachers that participated in our courses. This means that both groups get a comparable amount of Neuroscientific information. Of course the scientific stays are not easily scalable unless they are included as part of the regular teacher training (Ansari, 2021). Thus, the question remains about whether there are differential benefits of the two long-term strategies. We approached this question with the creation of an *ad hoc* questionnaire probing the epistemological and methodological knowledge of participants, adapting a set of questions we use in undergraduate courses. The responses show a great variability within groups, so only the group of graduate students shows a consistent difference with respect to the other groups. Nevertheless, the pattern of responses shows that some of the teachers that participated in the scientific stays tend to have a similar pattern of responses to the scientists and graduate students. In particular, notice that for the Neuromyth scale, the pattern of responses clearly separates in three groups, mainly driven by the response to the questions related to lateralization, the presence of excess stimulation in classrooms, the role of the brain in emotions and the lack of plasticity. More importantly, when a methodological questionnaire was applied, the questions related to experimental design were the most discriminating ones. These are in general hard questions in a sense that they are the core of scientific research and take some time to be deeply comprehended.

These are preliminary results, in particular because we used a convenience sample and the power might not be enough to detect other differences. If these results are confirmed in a controlled experiment, they would show that hands-on research activities can be an effective way to transmit the limits and implications of scientific research, allowing teachers to gauge the evidence and decide for themselves. In a way, this is the same that happens in Medicine, where medical doctors are supposed to read and interpret the findings of a wide range of disciplines to get a clearer picture of diagnoses and treatments. The same could happen in Education, where teachers should be prepared to critically assess the evidence coming from different sources, including Neuroscience. In a sense this is much more important than knowing specific bits of Neuroscientific knowledge. It is not impossible that some parts of our knowledge about Neuroscience change (for instance our ideas about learning and plasticity, Gallistel and King, 2009; Johansson et al., 2014). In fact, most epistemological considerations point to the possibility of change of scientific models and ideas. In that sense, the list of statements one should know would change and would require a constant updating. We subscribe the proposal, instead, that we should focus on educating the public in general, and teachers in particular, to be able to understand the design and methodology of studies involved in gathering relevant evidence for their fields (Pasquinelli, 2012; Ansari, 2021). This will surely require important amounts of declarative knowledge, but it should also include epistemological and methodological knowledge, which are probably better obtained by engaging in direct scientific activities. In order to confirm the relevance of this line of action we are starting a carefully controlled intervention that can test whether this is in fact the case or not.

## Data Availability Statement

The raw data supporting the conclusions of this article will be made available by the authors, without undue reservation.

## Ethics Statement

The studies involving human participants were reviewed and approved by Comité de Ética de la Investigación, Facultad de Psicología, Universidad de la República. The participants provided their consent to participate in this study, by accepting to enter the online form, after receving information about the study.

## Author Contributions

AC, AM, and JV-L conceived the study and designed the data collection steps. Most of the analysis has been done by JV-L. The results were thoroughly discussed by all authors. All authors participated equally in the writing of the manuscript.

## Funding

This work was funded by a grant from the Espacio Interdisciplinario (CICEA; 2021–2025), and ANII FSED-2-138821. This work was partially supported by PEDECIBA (Uruguay).

## Conflict of Interest

The authors declare that the research was conducted in the absence of any commercial or financial relationships that could be construed as a potential conflict of interest.

## Publisher's Note

All claims expressed in this article are solely those of the authors and do not necessarily represent those of their affiliated organizations, or those of the publisher, the editors and the reviewers. Any product that may be evaluated in this article, or claim that may be made by its manufacturer, is not guaranteed or endorsed by the publisher.

## Footnotes

^1^https://www.youtube.com/watch?v=O4xiApaSprM.

^2^https://www.fundaciontelefonica.uy/noticias/aula-en-linea-un-aporte-a-la-formacion-semipresencial-de-los-docentes/.

^3^This viewpoint was not known for most of educators in Uruguay at that time.

^4^https://www.youtube.com/playlist?list=PL_MnlbUI01SOU0193gpBrMJWAH692UujK.

^5^The 2nd symposium of education, cognition and neuroscience is scheduled for November of this year (2021) and we hope to have more than 500 Uruguay educators, but also from other countries of the region. See more details here: http://www.succc.org.uy/es/events/conference_2020.
