# Supplementary material for: Teaching the Science in Neuroscience to Protect From Neuromyths: From Courses to Fieldwork
Source: Front Hum Neurosci. 2021 Sep 28;15:718399. doi: 10.3389/fnhum.2021.718399 (PMC8506040; doi:10.3389/fnhum.2021.718399)
Supplement: Supplementary file 1 [file Data_Sheet_1.docx]

Supplementary Material

# 1. Questionnaire items

Here we present the items that we include in the survey. First (A) we present the items that make the Neuromyth scale and then (B) the ad hoc questions used to assess methodological knowledge.

# Items selected from Howard Jones et al (2009) survey and their adaptation to Spanish.

- 1. *The mind is the result of the action of the spirit, or of the soul, on the brain(I*).*

La mente es el resultado de la acción del espíritu, o del alma, en el cerebro.

- 2. *If there are ways to study brain activity, the mind can be studied through them(C*).*

La mente se puede estudiar a través del análisis y estudio de la actividad cerebral.

- 3. *The mind is a product of the working of the brain(C*).*

La mente es producto del funcionamiento del cerebro.

- 4. *Without a brain, consciousness is not possible(I*).*

Sin cerebro, la conciencia no es posible

- 5. *Intuition is a “special sense” that cannot be explained by the brain (I*).*

La intuición es un "sentido especial" que en principio no puede ser explicado por la actividad cerebral.

(*)Subjective opinion about the mind-brain relationship based on Howard Jones (2009) categorizations. I=incorrect; C=correct.

- 6. *One’s environment can influence hormone production and, in turn, personality* (C**)

El entorno puede influir en la producción de hormonas y, a la vez, en la personalidad.

- 7. *We use our brains 24 hours a day* (C**)

Usamos nuestro cerebro 24 horas al día

- 8. *To learn how to do something, it is necessary to pay attention to it* (C**).

Para aprender una habilidad compleja es necesario prestarle atención.

- 9. *Learning occurs through modification of the brain’s neural connections* (C**)

El aprendizaje ocurre mediante la modificación de las conexiones neuronales en el cerebro.

- 10. *Performance in activities such as playing the piano improves as a function of hours spent practising* (C**).

El desempeño en actividades como tocar el piano mejora en función de las horas dedicadas a la práctica.

- 11. *It is with the brain, and not the heart, that we experience happiness, anger, and fear* (C**).

Es con el cerebro, no con el corazón que experimentamos felicidad, ira y miedo.

- 12. *Memory is stored in the brain much like as in a computer. That is, each memory goes into a tiny piece of the brain* (I**)

La memoria se almacena en el cerebro al igual que en una computadora. Es decir, cada recuerdo entra en un pedacito del cerebro

- 13. *Memory is stored in networks of cells distributed throughout the brain* (C**).

La memoria se almacena en redes neuronales distribuidas en todo el cerebro.

- 14. *Keeping a phone number in memory until dialling, recalling recent events & distant experiences, all use the same memory system* (I**).

Mantener un número de teléfono en la memoria hasta marcar, recordar eventos recientes y experiencias distantes, todos usan el mismo sistema de memoria.

- 15. *When we sleep, the brain shuts down* (I**)

Cuando dormimos el cerebro se apaga.

- 16. *Learning is not due to the addition of new cells to the brain* (C**)

El aprendizaje no se debe mayoritariamente a la adición de nuevas células al cerebro.

- 17. *Emotional brain processes interrupt those brain processes involved with reasoning* (I**)

El procesamiento emocional interrumpe los procesos cerebrales relacionados con el razonamiento.

- 18. *Cognitive abilities are inherited and cannot be modified by the environment or by life experience* (I**)

Las habilidades cognitivas son heredadas y no pueden ser modificadas por el ambiente o por experiencia de vida.

- 19. *We mostly only use 10% of our brains* (I**)

La mayoría de las veces solo usamos el 10% de nuestro cerebro

- 20. *Children are less attentive after sugary drinks and snacks* (I**)

Los niños están menos atentos después de beber bebidas azucaradas y comer golosinas o “snacks”

- 21. *Environments that are rich in stimulus improve the brains of pre-school children* (I**)

Los entornos de clase ricos en estímulos mejoran el cerebro de los niños en edad preescolar

- 22. *Individuals learn better when they receive information in their preferred learning style* (e.g. visual, auditory, kinaesthetic) (I**)

Las personas aprenden mejor cuando reciben información en su estilo de aprendizaje preferido (por ejemplo, visual, auditivo, cinestésico)

- 23. *Short bouts of coordination exercises can improve integration of left and right hemispheric brain function* (I**)

Los períodos cortos de ejercicios de coordinación pueden mejorar la integración de la función cerebral hemisférica izquierda y derecha.

- 24. *Regular drinking of caffeinated soft drinks reduces alertness* (C**)

El consumo regular de refrescos con cafeína reduce el estado de alerta

- 25. *Differences in hemispheric dominance (left brain, right brain) can help explain individual differences amongst learners* (I**)

Las diferencias en la dominancia hemisférica (cerebro izquierdo, cerebro derecho) pueden ayudar a explicar diferencias individuales de desempeño entre los alumnos

- 26. *Learning problems associated with developmental differences in brain function cannot be remediated by education* (I**)

No es posible remediar los problemas de aprendizaje asociados con diferencias de desarrollo en la función cerebral.

- 27. *There are no critical periods in childhood after which you can’t learn some things, just sensitive periods when it’s easier* (C**)

No hay períodos críticos en la infancia después de los cuales no se pueda aprender algunas cosas, solo existen períodos sensibles cuando el aprendizaje es más fácil.

- 28. *Vigorous exercise can improve mental function* (C**)

El ejercicio vigoroso puede mejorar la función mental

- 29. *Individual learners show preferences for the mode in which they receive information (e.g. visual, auditory, kinaesthetic)* (C**)

Los alumnos muestran preferencias individuales sobre el modo en que reciben información (por ejemplo, visual, auditiva, cinestésica)

- 30. *Production of new connections in the brain can continue into old age* (C**)

La producción de nuevas conexiones cerebrales puede continuar hasta la vejez.

- 31. *Extended rehearsal of some mental processes can change the shape and structure of some parts of the brain* (C**).

La práctica extendida de algunos procesos mentales puede cambiar la forma y la estructura de algunas partes del cerebro.

- 32. Drinking less than 6-8 glasses of water a day can cause the brain to shrink (I**)

Beber menos de 6-8 vasos de agua al día puede hacer que el cerebro se encoja

(**) 6-32 General assertions (C=correct assertion, I=incorrect assertion) selected from Howard-Jones (2009).

# Ad hoc items to asses general epistemological investigation knowledge

1. Un investigador analiza relaciones entre muchas variables y la lectura y encuentra que aquellos niños que tienen mayor peso relativo, leen mejor y aquellos que tienen menos peso leen peor. El investigador demuestra así que incrementar el peso mejora la lectura (I).

*A researcher analyzes the relationship between a set of variables and reading scores. He finds that those children who have higher relative weight read better than those who have low weight. Therefore, the researcher shows that increasing weight improves reading.*

1. Una prueba de lectura al final de la Educación Media Superior muestra que la mayoría de los estudiantes que culmina tiene un buen vocabulario. Eso muestra que no es necesario buscar enriquecer el vocabulario de los jóvenes para que logren avanzar en la educación (I).

*A test applied to all students who finish High School shows that the majority of students has a good vocabulary knowledge. This shows that it is not necessary to try to enrich youngster’s vocabulary in order for them to advance in Education.*

1. Un investigador ganador del premio Nobel afirma que la técnica que desarrolló hace muchos años permite evaluar si una persona está infectada por un virus. Esto no demuestra que puede usarse la técnica para evaluar si una persona está infectada por un virus (C).

*A Nobel prize winning researcher claims that the technique he developed many years ago makes it possible to assess whether a person is infected by a virus. This does not show that the technique can be used to assess whether a person is infected with a virus.*

1. Un colegio usa un método para la enseñanza de la matemática, luego realiza una evaluación y todos sus estudiantes aprueban. No podemos afirmar que el método sea efectivo para la enseñanza de la matemática (C).

*A primary school applies a method for teaching math, then takes an exam and all of its students pass. We cannot claim that the method is effective for teaching mathematics.*

1. Un grupo de estudiantes realiza resúmenes de textos y les va bien en los exámenes. Esto prueba que hacer resúmenes es una buena manera de estudiar (I).

*A group of students summarize texts and perform well on tests. This proves that summarizing is a good way to study.*

1. Para evaluar un programa de alfabetización inicial se seleccionan niños al azar de diversas clases del país, dividiéndolos en dos grupos indistinguibles desde el punto de vista estadístico. Uno de los grupos realiza el programa nuevo y el otro un programa usual. Si los evaluadores externos encuentran ventajas estadísticamente significativas en aquellos niños que siguieron el programa nuevo, es posible afirmar que es más efectivo para la alfabetización inicial que el programa usual (C).

*In order to evaluate an initial literacy program, children are randomly selected from various classes in the country, dividing them into two statistically indistinguishable groups.One of the groups learn through the new program and the other through a regular program. If external evaluators find statistically significant advantages in those children who participated in the new program, it is possible to claim that it is more effective for initial literacy than the usual program.*

1. **PCA variance, loadings and items with the highest PCA loadings.**

**2.1. PCA, variance explained**

**2.1.a. In the neuromyth the percentage of variance explained by each of the first new dimensions (we only present up to 6 dimensions) is:**

| PC | 1 | 2 | 3 | 4 | 5 | 6 |
| --- | --- | --- | --- | --- | --- | --- |
| % variance | 15.8 | 8.7 | 7.8 | 6.3 | 5.8 | 5.3 |
| cummulated | 15.8 | 24.5 | 32.3 | 38.6 | 44.4 | 49.7 |

**2.1.b In the methodological scale the percentage variance explained by each of the first new dimensions is:**

| PC | 1 | 2 | 3 | 4 | 5 | 6 |
| --- | --- | --- | --- | --- | --- | --- |
| % variance | 36.5 | 18.5 | 15.7 | 15.5 | 8.6 | 5.1 |
| cummulated | 36.5 | 55.0 | 70.7 | 86.2 | 94.8 | 99.9 |

**2.2 The following tables display the most important items ranked in descending order according to the absolute loadings they have in the two PCA dimensions of each scale.**

**2.2.a. Neuromyth scale**

- - 1. First Principal Component. First five items.

| Ranking | Item | Loading |
| --- | --- | --- |
| 1 | *Short bouts of coordination exercises can improve integration of left and right hemispheric brain function.* | 0.40 |
| 2 | *Environments that are rich in stimulus improve the brains of pre-school children.* | 0.39 |
| 3 | *It is with the brain, and not the heart, that we experience happiness, anger, and fear.* | 0.35 |
| 4 | *Cognitive abilities are inherited and cannot be modified by the environment or by life experience.* | 0.31 |
| 5 | *Without a brain, consciousness is not possible.* | 0.30 |

- - 1. Second Principal Component. First five items.

| Ranking | Item | Loading |
| --- | --- | --- |
| 1 | *Learning problems associated with developmental differences in brain function cannot be remediated by education.* | 0.60 |
| 2 | *Learning is not due to the addition of new cells to the brain.* | -0.35 |
| 3 | *The mind is the result of the action of the spirit, or of the soul, on the brain.* | -0.28 |
| 4 | *We use our brains 24 hours a day.* | -0.26 |
| 5 | *We mostly only use 10% of our brains.* | -0.24 |

**2.2.b Most important items of the methods scale PCA**

1. First PC. The three most important items (out of 6).

| Ranking | Item | Loading |
| --- | --- | --- |
| 1 | *A primary school applies a method for teaching math, then takes an exam and all of its students pass. We cannot claim that the method is effective for teaching mathematics* | -0.67 |
| 2 | *A Nobel prize winning researcher claims that the technique he developed many years ago makes it possible to assess whether a person is infected by a virus. This does not show that the technique can be used to assess whether a person is infected with a virus.* | -0.66 |
| 3 | *A group of students summarize texts and perform well on tests. This proves that summarizing is a good way to study.* | -0.32 |

1. Second PC. The three most important items

| Ranking | Item | Loading |
| --- | --- | --- |
| 1 | *In order to evaluate an initial literacy program, children are randomly selected from various classes in the country, dividing them into two statistically indistinguishable groups.One of the groups learn through the new program and the other through a regular program. If external evaluators find statistically significant advantages in those children who participated in the new program, it is possible to claim that it is more effective for initial literacy than the usual program.* | -0.84 |
| 2 | *A group of students summarize texts and perform well on tests. This proves that summarizing is a good way to study.* | 0.31 |
| 3 | *A researcher analyzes the relationship between a set of variables and reading scores. He finds that those children who have higher relative weight read better than those who have low weight. Therefore, the researcher shows that increasing weight improves reading.* | -0.28 |
